# Screening of Multiple Myeloma by Polyclonal Rabbit Anti-Human Plasmacytoma Cell Immunoglobulin

**DOI:** 10.1371/journal.pone.0059117

**Published:** 2013-04-01

**Authors:** Bo Mu, Huan Zhang, Xiaoming Cai, Junbao Yang, Yuewu Shen, Baofeng Chen, Suhua Liang

**Affiliations:** 1 The Medical Biology Staff Room of North Sichuan Medical College, Sichuan Nanchong, the People’s Republic of China; 2 Sichuan Key Laboratory of Medical Imaging, Affiliated Hospital of North Sichuan Medical College, North Sichuan Medical College, Nanchong, the People’s Republic of China; University of Medicine and Dentistry of New Jersey, United States of America

## Abstract

Antibody-based immunotherapy has been effectively used for tumor treatment. However, to date, only a few tumor-associated antigens (TAAs) or therapeutic targets have been identified. Identification of more immunogenic antigens is essential for improvements in multiple myeloma (MM) diagnosis and therapy. In this study, we synthesized a polyclonal antibody (PAb) by immunizing rabbits with whole human plasmacytoma ARH-77 cells and identified MM-associated antigens, including enlonase, adipophilin, and HSP90s, among others, via proteomic technologies. 3-(4,5-Dimethylthiazol-2-yl)-2,5-diphenyltetrazolium bromide assay showed that 200 µg/mL PAb inhibits the proliferation of ARH-77 cells by over 50% within 48 h. Flow cytometric assay indicated that PAb treatment significantly increases the number of apoptotic cells compared with other treatments (52.1% vs. NS, 7.3% or control rabbit IgG, 9.9%). In vivo, PAb delayed tumor growth and prolonged the lifespan of mice. Terminal deoxynucleotidyl transferase dUTP nick end labeling assay showed that PAb also induces statistically significant changes in apoptosis compared with other treatments (P<0.05). We therefore conclude that PAb could be used for the effective screening and identification of TAA. PAb may have certain anti-tumor functions in vitro and in vivo. As such, its combination with proteomic technologies could be a promising approach for sieving TAA for the diagnosis and therapy of MM.

## Background

Multiple myeloma (MM), which accounts for approximately 10% of all malignant hematologic neoplasms [1], is difficult to cure by conventional chemotherapy, high-dose radiotherapy, autologous stem cell transplantation, and allogeneic transplantation [2], [3]. Immunotherapy based on antibodies has achieved significant success for MM treatment [4], [5]. Targeting of cell-surface antigens with promising monoclonal antibodies is a very attractive approach for treating MM. Rituximab, Daratumumab, atlezumab, and atlizumab [5]–[7] have been evaluated in preclinical and clinical studies. However, only a few tumor-associated antigens (TAAs) or therapeutic targets are currently available. Thus, identification of novel antigens is necessary to improve MM immunotherapy.

Over the last 20 years, several approaches have been used for the identification of TAA, among which serological screening of cDNA expression libraries, phage display libraries, and, more recently, proteomics-based approaches have been the most successful. Hundreds of candidate TAAs have been identified in various human cancer types [8], including liver cancer, breast cancer [9], prostate cancer [10], ovarian cancer [11], renal cancer [12], head and neck cancer [13], esophageal cancer [14], lymphoma [15], gastric cancer [16]and leukemia [17].

TAAs have been used mainly to identify tumor-specific overexpressing proteins in patient serum and/or tissue. The amount of certain TAAs in the circulation and/or tumor tissue is usually very low, especially during the early stages of cancer. In addition, antigens that are highly expressed in a tumor from a particular patient may not be overexpressed in a tumor from another patient. An example of such a TAA is CD20, which has been detected only in 13% to 22% of the patients studied [18]. TAA may also display heterogeneity in terms of epitope recognition within a given antigen. Thus, the current methods must be optimized continually to enhance the identification of candidate TAAs.

In the present study, we synthesized a polyclonal antibody (PAb), specifically anti-human MM line ARH-77 cells, and then screened and identified multiple proteins, including enolase, adipophilin (ADPH), and HSP90s, among others, as potential TAAs via proteomics-based approaches. Flow cytometric assay and immunofluorescence staining showed that the antigens are expressed in the ARH-77 cellular membrane. Verification of the antitumor functions of PAb showed the inhibitory effect of PAb on MM growth and its ability to induce apoptosis of myeloma cells in vitro and in vivo. Our results suggest that PAb may be effectively used for screening and identifying TAAs and that the PAb produced by the proposed method could have certain anti-tumor functions.

## Materials and Methods

### Animals and Cell Lines

SCID mice (6 wk to 8 wk old) were purchased from the Model Animal Research Center of Nanjing University. New Zealand white rabbits were purchased from the West China Experimental Animal Center. Animal protocols for the experiments were approved by the West China Hospital Cancer Center's Animal Care. In this study, two human MM ARH-77 and U266 cell lines and one human Burkitt's lymphoma Raji cell line obtained from the American Type Culture Collection were cultured in RPMI-1640 (Gibco BRL) containing 10% heat-inactivated FCS, 100 units/mL penicillin, and 100 units/mL streptomycin in a humid incubator with 5% CO_2_ at 37°C.

### Rabbit Immunization and PAb Isolation

PAb was generated by immunizing New Zealand white rabbits with ARH-77 cells with densities ranging from 1×10^7^ to 5×10^7^ cells per injection. The rabbits were then inoculated with Freund’s complete adjuvant (Sigma) followed by three booster injections of Freund’s incomplete adjuvant (Sigma) once every 10 d to 14 d. Sera were pooled after week administration of the last injection. Blood was allowed to clot overnight at 4°C, after which the serum was removed from the top of the mixture by centrifugation at 12000 *g*. Immunoglobulin (Ig) was isolated using an affinity chromatography system (AKTA Explore, GE, USA), freeze-dried using a freeze dryer (Rlphr 1–4 LSC, Christ, Germany), and kept frozen at −80°C until use. Control rabbit IgG was similarly purified from whole normal rabbit serum.

### Enzyme-linked Immunosorbent Assay (ELISA)

Tumor cells (5×10^3^ per well) were grown overnight in a poly-lysine-coated-96-well plate for ELISA. The media were removed and the cells were washed three times with PBS. After washing, the cells were blocked with 5% skim milk in blocking buffer (PBS containing 0.05% Tween-20, PBST) for 1 h at room temperature. The blocking reagent was then removed and cells were washed three times with PBS before addition of PAb. The PAb was diluted from 1∶2,000 to 1∶20,000 in dilution buffer (5% skim milk in PBST) and incubated for 1 h at room temperature. The antibody was then removed and the cells were washed three times with PBST. A second antibody (goat anti-rabbit linked to alkaline phosphatase, 1∶5,000, Sigma) was added to the cells and incubated for 30 min. The cells were then washed three times with PBST. Alkaline phosphatase substrate BCIP/NCP (Sigma) was subsequently added to the cells and the absorbance of the mixtures was measured at 450 nm using a 96-well plate reader (Molecular Device, M5, USA).

### Western Blot

Western blot was conducted as described previously [19]. Briefly, ARH-77 cells were lysed in 1 mL of lysis buffer. Proteins (25 µg/lane) were separated by SDS-PAGE and transferred on polyvinylidene fluoride (PVDF) membranes by electroblotting. The membranes were then blocked in 5% (w/v) skim milk, washed, and probed with PAb at 1∶20,00 dilution. Blots were washed and incubated with an HRP-conjugated secondary antibody diluted from 1∶5,000 to 1∶10,000 and visualized with chemiluminescence reagents.

### Immunofluorescence Staining of MM Cells

Three myeloma cell lines and two non-myeloma cell lines in the logarithmic phase were harvested and washed with PBS three times. The cells were blocked with 5% skim milk in PBST for 1 h at room temperature, after which the blocking reagent was removed. PAb and control rabbit IgG diluted to 1∶1,000 in PBST containing 5% skim milk were added to the cells. Incubation for 30 min followed. The antibody was then removed and the cells were washed three times in PBST. The second antibody (FITC-goat anti-rabbit IgG, 1∶500; Beijing Zhong Shan Golden Bridge Biological Technology Co., Ltd., China) was added to the cells. Incubation for 30 min followed. The antibody was then removed and the cells were washed three times in PBST. Up to 10,000 cells were acquired for flow cytometric analysis (Beckman-Coulter, USA).

### Localization of PAb Binding with Antigens on MM Cells

About 5×10^6^ cells were fixed with 100 µL 4% formaldehyde in PBS for 5 min at pH 7.6, after which 30 µL of the cell suspension was spread on a microscope slide by cell smearing. After drying, the cells were made permeable by treatment for 5 min with 0.5% Triton X-100/10 mM Hepes/300 mM sucrose/3 mM MgCl2/50 mM NaCl (pH 7.4) and incubated with PAb or control IgG (dilution 1∶1,000) overnight at 4°C. The antibody was then removed and the cells were washed three times in PBST. A second antibody (FITC-goat anti-rabbit IgG 1∶500; Beijing Zhong Shan Golden Bridge Biological Technology Co.) was added to the cells and the cells were incubated in a humidified chamber for 30 min. The antibody was removed and the cells were washed three times in PBST, stained with Hoechst33258 for 5 min, and then washed with PBS. Fluorescent microscopy was performed with a Zeiss Photoscope Imager Z I.

### Two-dimensional Electrophoresis (2D–E)

About 2×10^7^ ARH-77 cells were solubilized in 1 mL of lysis solution (7 M urea, 2 M thiourea, 4% CHAPS, 2 mmol/L TBP, 0.2% ampholyte, traces of bromophenol blue) at 4°C for 20 min. Insoluble material was removed by centrifugation at 15000 rpm at 4°C for 30 min. Protein concentrations were determined by the Bradford method. Samples were frozen at −70°C and thawed immediately before use. Approximately 1 mg protein was loaded on 17 cm of IPG Ready Strips. After rehydrating the strips for 14 h, IEF was carried out for 1 h at 200 V, 1 h at 500 V, and 1 h at 1000 V. A gradient was then applied from 1,000 to 8,000 for 1 h and finally at 8,000 V for 8 h to reach a total of 72 KVh at 20°C. After IEF separation, the gel strips were incubated first in equilibration buffer (50 mM Tris-HCl, pH 8.8, 6 M urea, 30% glycerol, 2% SDS) with 10 mg/mL DTT and then in equilibration buffer with 25 mg/mL iodoacetamide for 15 min each. The strips were then loaded on 12% SDS-PAGE gel and electrophoresed for 20 min at a constant current of 10 mA and at 30 mA per gel until the bromophenol blue indicator reached the bottom of the gels. One gel was then stained with Coomassie Brilliant Blue R-250 and destained with 40% methanol and 10% acetic acid. Another gel was analyzed by 2D Western blot.

### 2D Western Blot

The separated proteins were transferred on PVDF membranes and incubated for 2 h at room temperature with a blocking buffer consisting of TBST (Tris-buffered saline +0.01% Tween 20) and 5% skim milk. The PVDF membranes were dyed with Commassie Blue staining solution for 15 min [0.1% Coomassie Brilliant Blue R-250 (w/v) and 50% methanol (v/v)] and outstanding points were marked as landmarks. The membranes were then decolorized for 1 h in destaining solution [40% methanol (v/v) with 10% acetic acid (v/v)], washed, and incubated with PAb for 1 h at room temperature. After additional washes with TBST, the membranes were incubated with a secondary antibody conjugated with horseradish peroxidase at 1∶5,000 dilution for 1 h and transferred to Vectastain ABC (Vector Laboratories, Burlingame, CA, USA). The protein spots on the film were matched with the 2-DE map of the same sample and excised from the 2-DE gel stained with Coomassie Brilliant Blue. The excised proteins were then digested as described previously for protein identification by mass spectrometry using spots from different gel with at least two replicates. The obtained peptide mass fingerprint was used to search through the Swiss-Prot and National Center for Biotechnology Information nonredundant databases by the Mascot search engine (www. matrixscience.co.uk). Protein identification was reconfirmed by an ESI-MS/MS approach. The database search was finished with the Mascot search engine (www.matrixscience.co.uk) using a Mascot MS/MS ion search.

### Cell Viability Analysis by 3-(4,5-dimethylthiazol-2-yl)-2, 5-diphenyltetrazolium Bromide (MTT) Assay

Cell growth inhibition was determined by MTT assay (Sigma). Briefly, myeloma cells (2×10^4^ cells/well to 3×10^4^ cells/well) were seeded on a 96-well plate at a volume of 100 µL per well and incubated for 24 h. The cells were then treated with normal saline (NS), control rabbit IgG, or PAb for 48 h at 37°C and subjected to MTT assay. Cells treated with NS served as the indicator of 100% cell viability.

### Apoptosis Analysis by Flow Cytometry Assay

The cells (3×10^5^ per well) were plated in 6-well plates and treated with NS, control IgG (200 µg/mL) or PAb (200 µg/mL). After 48 h, flow cytometric analysis was conducted to identify sub-G1 phase cells/apoptotic cells. Briefly, the cells were suspended in 1 mL of hypotonic fluorochrome solution containing 50 µg/mL propidium iodide in 0.1% sodium citrate with 0.1% Triton X-100 and analyzed by a flow cytometer. Apoptotic cells appeared in the cell cycle distribution as cells with DNA content less than that of G1 phase cells.

### Antitumor Effect of PAb on Xenograft SCID Mouse Models with ARH-77

Human ARH-77 MM cells (5×10^6^) were implanted subcutaneously into the right flanks of female SCID mice. When the tumor nodules were palpable, the mice were divided randomly into three groups with six mice each and treated with NS, control IgG, or PAb via the tail vein. Control IgG and PAb (200 µg/dose, dissolved in NS) were administered seven times every 2 d in a volume of 100 µL along with the control injection in a volume of 100 µL NS. The tumor volume was observed and the tumor size was determined once every 3 d by caliper measurement as described previously [20].

### Terminal Deoxynucleotidyl Transferase-mediated dUTP Nick end Labeling (TUNEL) Assay

Cell apoptosis in vivo was examined by TUNEL assay according to the manufacturer’s instructions (Promega, USA). Three tumors per group were analyzed 48 h after the last treatment.

### Statistical Analysis

SPSS version 13 was used for statistical analysis. The statistical significance of results in all of the experiments was determined by Student’s t-test and analysis of variance. The findings were regarded as significant if P<0.05.

## Results

### Production and Characterization of PAb

**Figure 1:**
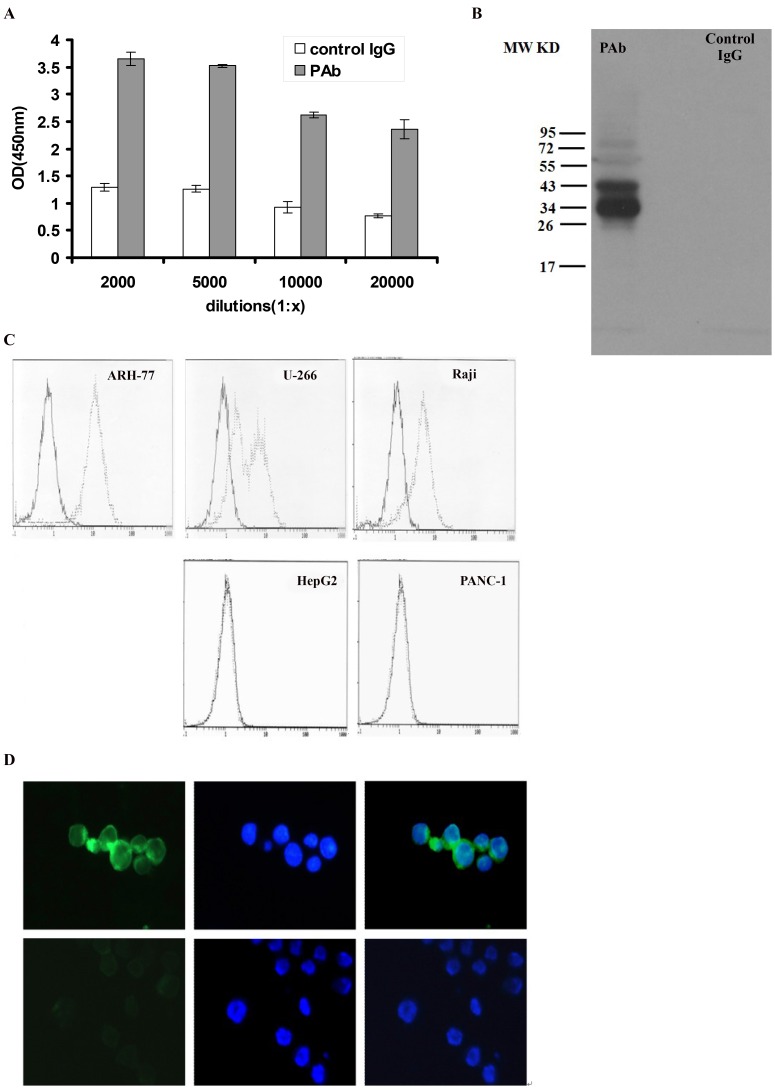
Production and characterization of PAb. (A) ELISA of PAb on ARH-77. Control rabbit IgG and PAb were incubated with ARH-77 at dilutions from 1∶2,000 to 1∶20,000. After addition of an alkaline phosphatase-conjugated secondary antibody, the absorbance was measured at 450 nm. Represented here is the mean of 4 wells to 6 wells ± standard deviation for every dilution. (B) Western blot showed the multiple protein bands recognized by PAb but not by control IgG. (C) Indirect immunofluorescence assay of PAb on myeloma and non-myeloma cell line by flow cytometry. Gray line represents 1∶2,000 PAb dilutions reacted with ARH-77 (left panel), U266 (upper part, middle panel), and Raji (upper part, right panel), human hepatocellular carcinoma cell line HepG2 (lower part, middle panel) and human pancreatic carcinoma cell line Panc-1(lower part, right panel). Black line represents control IgG diluted to 1∶2,000 used as a negative control. (D) Indirect immunofluorescence assay of antigens on ARH-77 by fluorescence microscopy with FITC-goat anti-rabbit IgG (left, green fluorescence) and with hoechst33258 (middle, blue fluorescence). Up line represents the treatment group with PAb and down line represents the treatment group with control IgG. Merged images (right) show localization of antigens on ARH-77 cells (400×).

To investigate the possibility of vaccination of rabbits with ARH-77, two rabbits were inoculated with ARH-77 cells to produce polyclonal antibody. PAb was tested for its ability to bind MM cell lines (Fig. 1A). The binding of ARH-77 by PAb differed by 3- to 10-fold from control IgG. The binding was dose-dependent, with dilutions of 1∶2,000 and 1∶5,000 showing greater binding to ARH-77 than dilutions of 1∶10,000 or 1∶20,000. As to the antigens recognized by PAb, we further performed Western blot, flow cytometric assay, and immunofluorescence studies. ARH-77 cell lysates were probed with either PAb or control IgG on Western blots. Multiple bands (Fig. 1B) were recognized by PAb but not by the control IgG. Immunofluorescence and flow cytometric assay studies to detect the combination between PAb or control IgG and fixed ARH-77cells revealed that PAb significantly binds to the surface of ARH-77 but shows no reactivity to control IgG, as well as in U266 and Raji cells. However, PAb did not bind non-myeloma cell lines, such as the human hepatocellular carcinoma cell line HepG2 and human pancreatic carcinoma cell line Panc-1(Figs. 1C and 1D). These results suggest the synthesis of PAb with high specificity and ability to identify myeloma cell surface antigens.

### Antigen Identification of PAb by 2-DE/Western Blot and MALDI-TOF MS/MS Analysis

**Figure 2:**
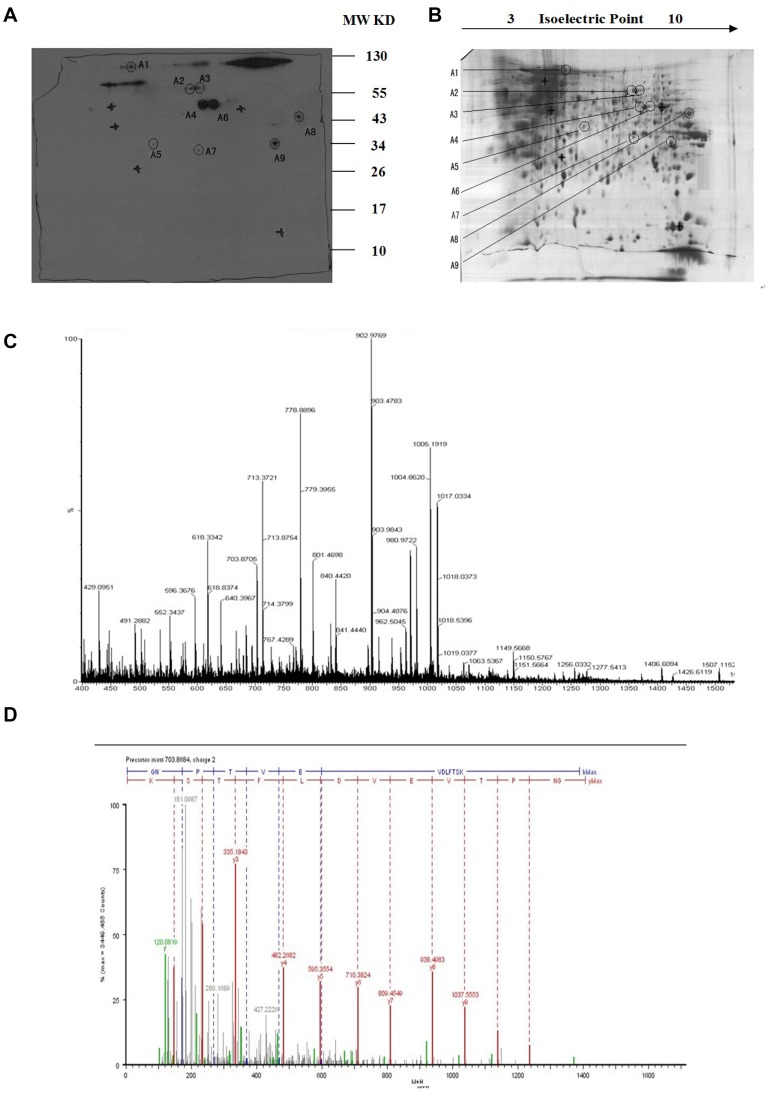
2-D PAGE and Western blot analysis of ARH-77 cell proteins. (A) Western blot detection of the targeted-protein spot recognized by PAb. (B) 2-D protein pattern of ARH-77 cells after Commassie Blue staining. (C) MALDI-MS spectrum obtained from spot A1 after trypsin digestion and peptide sequences from ENO1 matching peaks obtained from MALDI-MS spectra. (D) The peptide of 703.6864 selected from the PMF of the A1 spot was sequenced by nano-ESI-MS/MS.

To recognize the targeted antigens of PAb, 2-DE and Western blot were performed with the ARH-77 cell lysate. Gels (17 cm) (3 to 10 NL) were used for Western blot to determine the PI and MW of the corresponding antigens of PAb (Fig. 2A). The protein spot showing a positive reaction with PAb in X-film was excised from the gel (Fig. 2B). The excised gel piece was destained and trypsinized into peptides for MS and MS/MS analysis. Mass spectra were acquired with a Q-TOF Premier mass spectrometer. MS/MS data, including the mass values, the intensity, and the charge of the precursor ions, were analyzed with a licensed copy of the Mascot 2.0 program against the SWISS-PROT protein database. On the map, nine tumor-specific spots were excised and subjected to in-gel digestion followed by peptide mass fingerprinting for protein identification. Figure 2C shows the identification of Spot No.1 as an example. The results of antigen identification are summarized in the Appendix, Table 1.

**Table 1:** Protein spots in GC searched by Peptident software in the SWISS-PROT database.

Spot	Protein name	IPI: ID	Top score	Theoretic pI	Theoretic Mr	Sequence coverd Rate(%)
A1	Heat shock protein HSP 90-alpha (**HSP90A**)	IPI00382470	429	4.94	84607	35
A2	Stress-induced phosphoprotein 1 (**STIP1**)	IPI00013894	179	6.4	62599	23
A3	Bifunctional purine biosynthesis protein PURH (**PUR9**)	IPI00289499	205	6.27	64575	31
A4	Alpha-enolase (**ENO1**)	IPI00465248	1533	7.01	47139	45
A5	Adipophilin (**ADPH**)	IPI00293307	154	6.34	48045	28
A6	Vacuolar protein sorting-associated protein 37B (**VP37B**)	IPI00002926	50	6.78	31287	30
A7	Isocitrate dehydrogenase [NAD] subunit alpha (**IDH3A**)	IPI00030702	638	6.47	39566	26
A8	Phosphoglycerate kinase 1(**PGK1**)	IPI00169383	688	8.3	44586	45
A9	Voltage-dependent anion-selective channel protein 2 (**VDAC2**)	IPI00024145	158	7.49	31547	26

### Inhibitory Effect of PAb on ARH-77 Cell Proliferation

**Figure 3:**
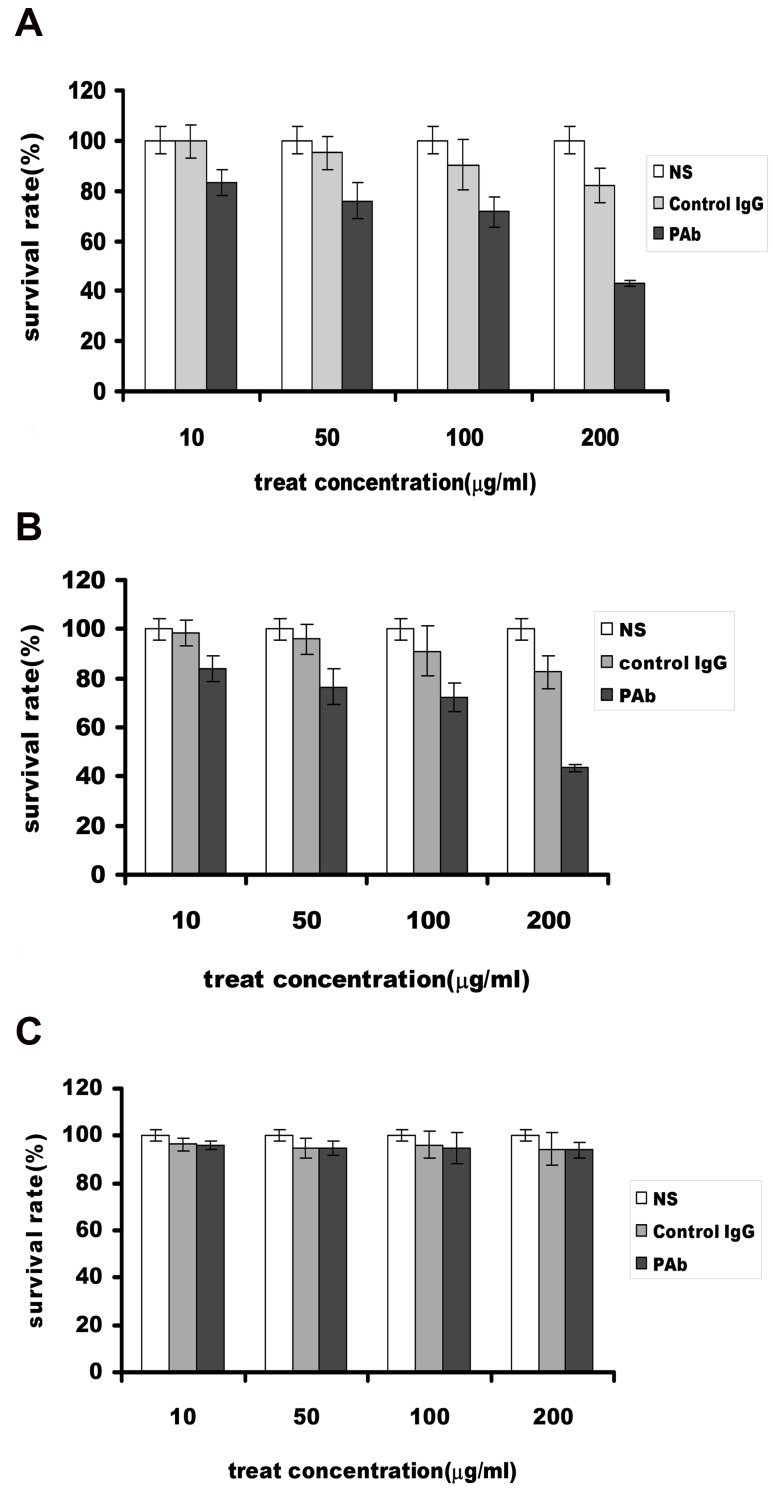
Inhibition of myeloma cells growth in vitro determined by MTT. (A)The growth of PAb-treated cells was significantly inhibited compared with the control IgG and NS groups, and the inhibitory rates on different concentrations on ARH-77 cells after 48 h were 16.7%, 23.98%, 28.47%, and 56.84%. (B)The similar results were shown in U266 cell line. (C) The PAb did not effect growth of HepG2 cell line.

**Figure 4:**
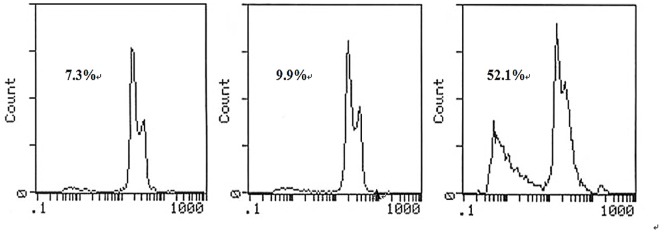
PAb-induced apoptosis in myeloma cell lines. Flow cytometric analysis revealed the proportion of sub-G1 phase cells (apoptotic cells) to be 7.3% (NS), 9.9% (control), and 52.1% (PAb). The experiments were repeated at least three times.

Antigens recognized by PAb, such as enolase, ADPH, and HSP90, were correlated closely with cancer cell proliferation, survival, and metastasis. Thus, we hypothesized that PAb could have antitumor functions for blocking these TAAs. The effect of PAb on the proliferation of ARH-77 cells was evaluated by MTT and flow cytometric assay. Compared with the NS and control IgG treated cells, the inhibitory rates of PAb in 10 µg/mL, 50 µg/mL, 100 µg/mL, and 200 µg/mL ARH-77 cells after 48 h were 16.7%, 23.98%, 28.47%, and 56.84%, respectively (Fig. 3A), and similar results were also shown in U266 cell lines (Fig. 3B), but the PAb did not effect the growth of HepG2 cell (Fig. 3C). The results indicate that PAb can decrease the proliferation of ARH-77 cells in vitro. Moreover, flow cytometric assay revealed that PAb treatment significantly increased the number of apoptotic cells compared with the other treatments (52.1% vs. NS, 7.3% or control IgG, 9.9%)(P<0.05; Fig. 4). These findings suggest that PAb inhibits proliferation and induces apoptosis in cancer cells in vitro.

### Inhibition of Tumor Growth in an Animal Model of Myeloma

**Figure 5:**
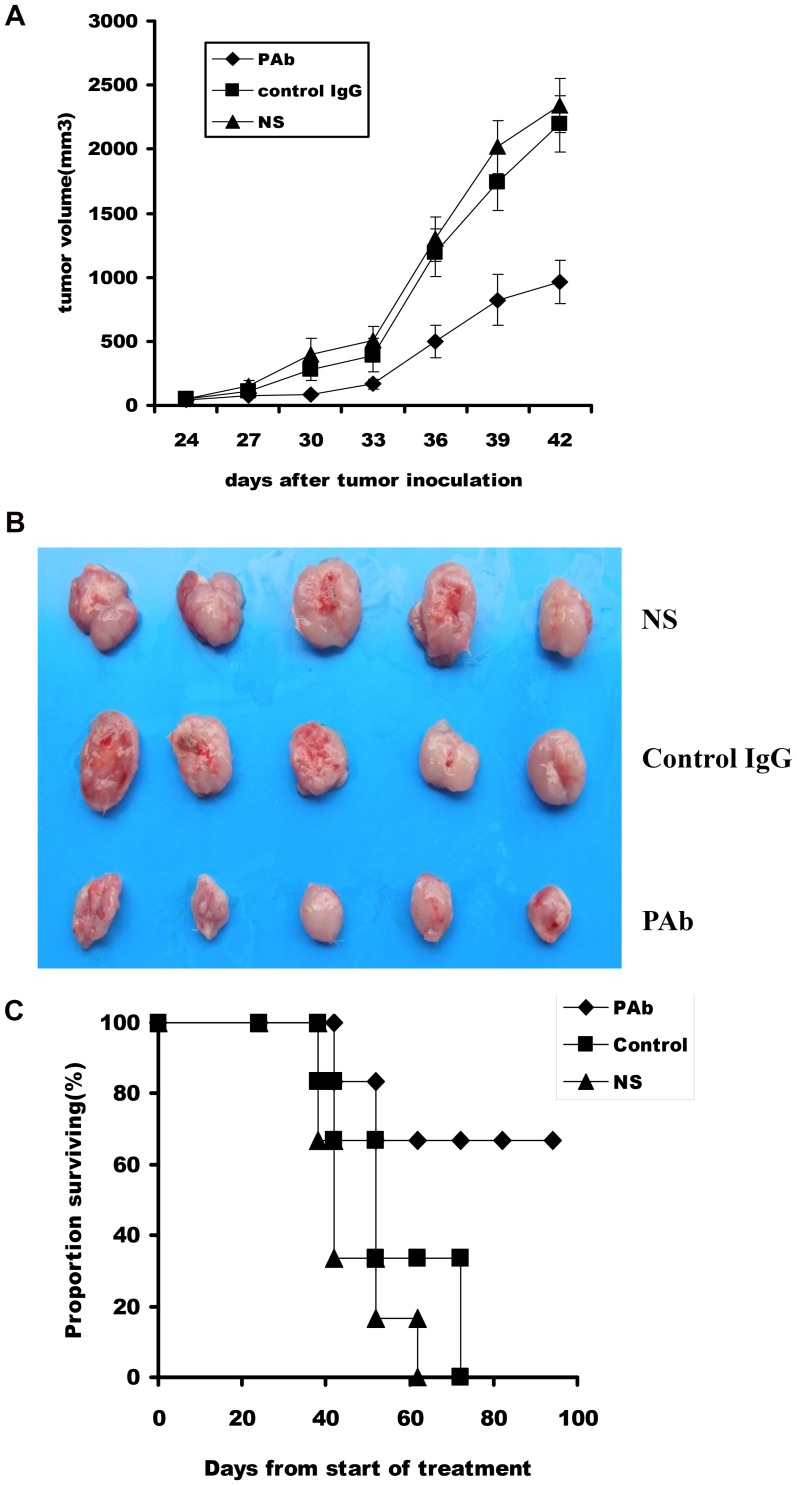
Inhibitory effect of PAb on tumor growth in xenograft SCID mouse models. (A) A significant difference in tumor volume (P<0.05) was observed between PAb-treated mice and other treatment groups. The mean ± standard error of the mean of tumor growth of five mice is shown. (B) Representative picture for tumor volume different groups. (C) A significant increase in survival was observed in PAb-treated mice compared with other treatment groups (P<0.05).

**Figure 6:**
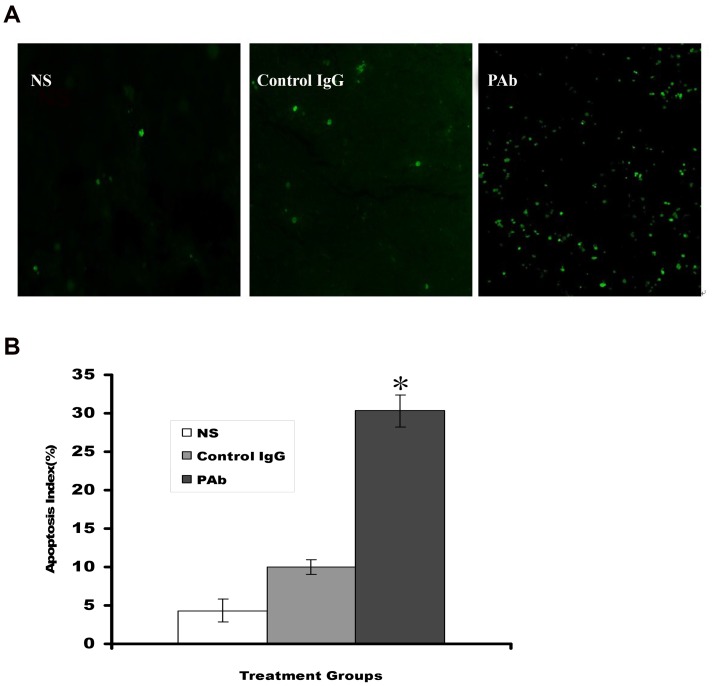
PAb-induced tumor cells apoptosis in vivo by TUNEL assay. (A) Sections from the tumor-bearing mice treated with NS (left panel), control IgG (middle panel), or PAb (right panel) were stained with FITC-dUTP as described in the Materials and Methods section (200×). (B) An apparent increase in the number of apoptotic cells and apoptotic index was observed within residual tumors treated with PAb compared with other treatment groups in the ARH-77 subcutaneous injection tumor models. * represents the PAb group showing significant difference compared with NS and control IgG group mice (P<0.05).

Based on the findings in vitro, we tested the efficacy of the PAb in an SCID mice model. The results show that PAb treatment significantly regresses the established tumors and prolongs the lifespan of mice compared with the NS or control IgG treatments (Fig. 5). The average tumor volume in the PAb group was stable for most of the experiment after administration of PAb whereas the average tumor volumes in NS and control IgG groups continued to increase. The inhibition rate reached approximately 61.6% in the PAb group. This result supports the hypothesis that PAb displays antitumor activity in vivo. TUNEL assay showed an apparent increase in the number of apoptotic cells and apoptotic index within the residual tumors treated with PAb compared with the NS and control IgG groups (P<0.05; Fig. 6). These data suggest that both inhibition of myeloma proliferation and apoptosis-inducing activity are involved in the antitumor effects of PAb.

## Discussion

The availability of high throughput 2-DE gels and initial screening using automated procedures has made the identification of TAA in the proteome of various tumor cell lines and/or tissues possible. This study was based on PAb combined with proteomic analysis and aimed to screen TAAs in the proteome level to help further improve the diagnosis and immunotherapy of MM. We synthesized a PAb by immunizing rabbits with the human plasmacytoma cell line ARH-77 and identified multiple TAAs of MM, such as enolase, ADPH, and HSP90s, among others, using 2-DE, Western blot, and mass spectrometric techniques.

To validate the MS/MS results, we selected three proteins for examination according to their positions in the Mascot score list, which lists the vital role they play in many cancers. These proteins are discussed below.

### α-Enolase

The propensity for glycolysis is enhanced in cancer cells because of increased cell proliferation. Previous studies have indicated that α-enolase, a key enzyme in the glycolysis pathway, is upregulated in 18 out of 24 types of cancer, as determined by bioinformatics study using gene chips and EST databases [21]. A recent proteomic analysis further revealed that overexpression of α-enolase in hepatitis C virus-related hepatocellular carcinomas is associated with tumor progression [22]. Although the mechanisms of the surface expression and orientation of α-enolase on the membrane have yet to be clearly understood, surface α-enolase is known to act as a strong plasminogen-binding receptor [23]. The binding of plasminogen to the cell surface and its consequent activation to plasmin may play crucial roles in the intravascular and pericellular fibrinolytic systems, cell invasion, tumor cell migration, and metastasis as a plasminogen-binding receptor [24]. Thus, we hypothesize that α-enolase is a diagnostic marker and therapeutic target of MM.

### ADPH

ADPH, a member of the perilipin family of lipid droplet-associated proteins, hypothetically mediates milk lipid formation and secretion [25]. Previous studies have indicated that ADPH functions in lipid storage droplets formation [26], fatty acid uptake [27], and milk lipid secretion [28]. In addition, ADPH is reportedly overexpressed in colorectal cancer [29], hepatocellular cancer, renal cell cancer [30]and kidney cancer [31].

To date, the direct interactions of ADPH with cancer cells have yet to be clearly understood. Cellular levels of ADPH are reportedly correlated with lipid accumulation in various cells and tissues [26], [32]. Moreover, ADPH is involved in lipid droplet/apical cell surface membrane recognition or interaction because of its interaction with milk lipid globule membranes inner surface coat constituents [33]. The recent discovery of human cancer cells expressing high levels of fatty acid synthase and undergoing significant endogenous fatty-acid synthesis has allowed researchers to perform in-depth reviews of the roles of fatty acids in tumor biology [34], [35]. Wright et al. [36] showed that ADPH could induce PPAR-gamma activation, which is a potential path for promoting tumor cell differentiation in malignant melanoma. ADPH can augment tumor-necrosis factor-α (TNF-α), MCP-1, and interleukin-6 (IL-6) expression [37]. However, IL-6 and TNF-α could mediate MM growth, survival, and resistance to apoptosis [38]. Thus, ADPH may be a novel target pathway for tumor therapy because of its interaction with cytokines and fatty acid synthesis.

### HSP90

HSP90, one of the most abundant molecular chaperones, is important for the maturation, stability, and activity of numerous cancer-related proteins, such as mutated p53, EarB2/Her2, Raf-1, cyclin-dependent kinases 1 and 4, Akt/PKB, Bcr-Abl, and Hif-1a, which are involved in cell signaling, proliferation, and survival, as well as neoangiogenesis, adhesion, and drug resistance [39], [40]. HSP90 is frequently overexpressed and activated in cancer cells, including acute leukemias [41], gastrointestinal cancers [42], glioblastoma [43], cervical cancer [44], lung cancers [45] and human breast cancers [46]. Several studies have shown that HSP90 is localized in the cytoplasm and on the cell surface in certain types of cancer cells [47], including prostate cancer [48], melanomas [49], non-small-cell lung cancer cells [50], fibrosarcoma cells [51], lymphomas [52] and breast cancer cell [53].

The mechanism of HSP90 function has been reviewed in detail [54]–[57] but knowledge of the function of cell-surface HSP90 in tumor cells is limited. HSP90 has been correlated with cancer metastasis [58]and migration of malignant cells [43]. HSP90 proteins may interact with other cell-surface proteins through transmembrane signaling, thereby triggering intracellular events necessary for cell invasion [47]. In addition, the cancer-specific expression of cell-surface HSP90 has been found to be associated with MHC class I [59] and increases in expression level through several stages of early and late apoptotic death with immune response activation [60]. Increasing evidence suggests that HSP90 can function as a central regulator of proliferative and anti-apoptotic signal transduction and may be a potential biomarker and therapeutic target for the immunotherapy of tumors like MM.

Other proteins have been implicated in cell proliferation [61], aging [62], multidrug resistance [63], [64], and mitochondrial apoptosis [65]. Although the data are preliminary, the antigens detected in this paper may be candidate diagnostic markers and therapy targets in MM. Proteomic technologies provide a powerful tool for identifying TAAs and, especially, verifying cellular membrane antigens.

The PAb produced by our method has certain antitumor functions in vitro and in vivo that may block TAAs correlated with tumor cell proliferation, survival, and apoptosis. We found that PAb induces the apoptosis of various myeloma cell lines, inhibits tumor growth, and prolongs the lifespan of mice. PAb also induces statistically significant apoptosis compared with other treatments, as determined by TUNEL assay in vivo. As such, its combination with polyclonal rabbit anti-human plasmacytoma cell globulins may be a promising approach for sieving TAA for the diagnosis and therapy of MM.

In conclusion, the use of PAb against whole tumor cells coupled with high throughput proteomic technologies could be a potential tool for screening TAAs. The major advantages of this approach are as follows: (1) the isolated antibodies bind to native forms of their antigens or ligands on the cell surface, whereas purified tumor antigens are often recombinant in nature and lack post-translational modification; (2) the antigens are accessible to the isolated antibodies, due to those isolated by using whole tumor cell as antigens could recognize multiple antigens at the same time; and (3) PAb can recognize multiple cell surface proteins correlated with cell death simultaneously. Thus, the approach linking high-throughput proteomic technologies can help discern TAAs efficiently. In the present study, we identified multiple proteins as TAA-presumed potential biomarkers and therapeutic targets for the immunotherapy of MM, including enolase, ADPH, and HSP90s, among others. To explore the function of these proteins and evaluate their clinical applicability and specificity, further studies need to be conducted Integration of PAb with target identification by proteomic technologies may ultimately translate into novel and improved diagnosis and therapy for cancer patients.
